# Low-dose lithium adjunct to atypical antipsychotic treatment nearly improved cognitive impairment, deteriorated the gray-matter volume, and decreased the interleukin-6 level in drug-naive patients with first schizophrenia symptoms: a follow-up pilot study

**DOI:** 10.1038/s41537-023-00400-w

**Published:** 2023-10-14

**Authors:** Chuanjun Zhuo, Shuiqing Hu, Guangdong Chen, Lei Yang, Ziyao Cai, Hongjun Tian, Deguo Jiang, Chunmian Chen, Lina Wang, Xiaoyan Ma, Ranli Li

**Affiliations:** 1 Key Laboratory of Sensor Information Processing Abnormalities in Schizophrenia (SIPAS-Lab), Tianjin Fourth Center Hospital, Nankai University Affiliated Tianjin Fourth Center Hospital, Tianjin Medical University Affiliated Tianjin Fourth Center Hospital, Tianjin, 300140 China; 2 Department of Psychiatry, Wenzhou Seventh Peoples Hospital, Wenzhou, 325000 China; 3 Laboratory of Psychiatric-Neuroimaging-Genetic and Co-morbidity (PNGC_Lab), Nankai University Affiliated Tianjin Anding Hospital, Tianjin Medical University Affiliated Tianjin Anding Hospital, Tianjin Mental Health Center of Tianjin Medical University, Tianjin Anding Hospital, Tianjin, 300222 China

**Keywords:** Neuroscience, Schizophrenia

## Abstract

This study was conducted to investigate the effects of long-term low-dose lithium adjunct to antipsychotic agent use on the cognitive performance, whole-brain gray-matter volume (GMV), and interleukin-6 (IL-6) level in drug-naive patients with first-episode schizophrenia, and to examine relationships among these factors. In this double-blind randomized controlled study, 50 drug-naive patients with first-episode schizophrenia each took low-dose (250 mg/day) lithium and placebo (of the same shape and taste) adjunct to antipsychotic agents (mean, 644.70 ± 105.58 and 677.00 ± 143.33 mg/day chlorpromazine equivalent, respectively) for 24 weeks. At baseline and after treatment completion, the MATRICS Consensus Cognitive Battery (MCCB) was used to assess cognitive performance, 3-T magnetic resonance imaging was performed to assess structural brain alterations, and serum IL-6 levels were quantified by immunoassay. Treatment effects were assessed within and between patient groups. Relationships among cognitive performance, whole-brain GMVs, and the IL-6 level were investigated by partial correlation analysis. Relative to baseline, patients in the lithium group showed improved working memory, verbal learning, processing speed, and reasoning/problem solving after 24 weeks of treatment; those in the placebo group showed only improved working memory and verbal learning. The composite MCCB score did not differ significantly between groups. The whole-brain GMV reduction was significantly lesser in the lithium group than in the placebo group (0.46% vs. 1.03%; *P* < 0.001). The GMV and IL-6 reduction ratios correlated with each other in both groups (*r* = −0.17, *P* = 0.025). In the lithium group, the whole-brain GMV reduction ratio correlated with the working memory improvement ratio (*r* = −0.15, *P* = 0.030) and processing speed (*r* = −0.14, *P* = 0.036); the IL-6 reduction ratio correlated with the working memory (*r* = −0.21, *P* = 0.043) and verbal learning (*r* = −0.30, *P* = 0.031) improvement ratios. In the placebo group, the whole-brain GMV reduction ratio correlated only with the working memory improvement ratio (*r* = −0.24, *P* = 0.019); the IL-6 reduction ratio correlated with the working memory (*r* = −0.17, *P* = 0.022) and verbal learning (*r* = −0.15, *P* = 0.011) improvement ratios. Both treatments implemented in this study nearly improved the cognitive performance of patients with schizophrenia; relative to placebo, low-dose lithium had slightly greater effects on several aspects of cognition. The patterns of correlation among GMV reduction, IL-6 reduction, and cognitive performance improvement differed between groups.

## Introduction

Schizophrenia is characterized by cognitive impairment, which has major impacts on functional outcomes, in about 98% of cases^1–6^. This impairment can be broad in scope, affecting individuals’ executive functions and related processes, thereby substantially compromising their ability to plan, reason, solve problems, and think abstractly^7^. Many strategies have been proposed to rescue cognitive impairment in patients with schizophrenia^8–11^. Cognitive impairment in patients with schizophrenia has been related to gray-matter volume (GMV) reduction in key brain regions^12–19^ and increased interleukin-6 (IL-6) levels^20,21^.

GMV reduction is a typical structural change in patients with various neuropsychiatric disorders, resulting from the alteration of synaptic pruning and microglial and astrocytic function, and usually associated with cognitive impairment^15,22^. For example, Zierhut et al. ^12^ reported that GMV reductions in the temporal lobe and mediofrontal cortex were associated with the impairment of working memory and other cognitive dimensions.

High levels of the inflammatory factor IL-6 have been associated with cognitive impairment in first-episode and high-risk schizophrenia cases^21,23–34^. IL-6 affects neuronal transmission and survival in the prefrontal cortex and hippocampus and causes the reduction of gray-matter thickness, and thus cognitive impairment, in patients with schizophrenia and other mental disorders^26,27,34–41^ Several therapeutic agents, including antipsychotics, reduce the IL-6 level and thus alleviate this effect^25,42–47^. However, the ability of IL-6 and its receptor to cross and increase the permeability of the blood–brain barrier, enabling the entry of other inflammatory agents, may contribute to poor functional outcomes and treatment resistance in patients with schizophrenia^31,48,49^. Several mechanisms underlying these effects have been proposed^50–54^, but few studies have involved the examination of associations among IL-6 levels, GMV alterations in the frontal lobe and hippocampus, and cognitive impairment in such patients.

Lithium can reduce the oxidative inflammatory response, at least in part through the inhibition of glycogen synthase kinase-3β (GSK-3β) expression; this effect contributes to its efficacy in the treatment of mood disorders^27,31,43,44,55–58^. Through its effect on GSK-3β, lithium reduces transcription factor activity, thereby reducing the production of pro-inflammatory mediators [including interferon (IFN)-γ and IL-6] and increasing that of anti-inflammatory cytokines^58–61^; GSK-3β inhibition has the opposite effects^61–64^. Lithium’s inhibition of GSK-3β also leads to the reduction of pro-inflammatory cytokine production via the reduced activation of the signal transducer and activator of transcription^62,63^. In patients with bipolar disorder, 30- and 90-day lithium regimens reduced the levels of inflammatory markers, including IL-6^58,64,65^. Similarly, an ex vivo study showed that lithium reduced the IL-6 production of lipopolysaccharide-stimulated monocytes from patients with bipolar disorder ex vivo^66–76^. Moreover, the effects of lithium on GSK-3β cascade (AKT/FoxO3a/β-catenin, AKT/GSK-3β/β-catenin, oxidative stress, and inflammatory factor pathway) activity are neuroprotective. These cascades play critical roles in brain development and were found to impair cognitive function in a murine model of schizophrenia, and their inhibition enhances frontal-lobe neural activity, improving cognitive function^67,68,70,75,76^. Lithium also improves cognitive performance by increasing the cortical N-acetyl-aspartate concentration^69,74^.

This evidence converges to support the association of cognitive impairment in schizophrenia with GMV reduction and increases in the IL-6 level. Lithium improves such impairment by regulating IL-6. Exploration of the relationships among these factors will provide useful information guiding the development of strategies to reduce cognitive impairment in schizophrenia, especially first-episode schizophrenia in drug-naive patients. In this double-blind randomized controlled study, we used an IL-6 immunoassay, 3.0-T magnetic resonance imaging (MRI), and the MATRICS Consensus Cognitive Battery (MCCB) to investigate the associations among the IL-6 level, GMV alterations, and cognitive impairment in drug-naive patients with first-episode schizophrenia. We hypothesized that: 1) a 24-week regimen of low-dose lithium adjunct to antipsychotic agent use would improve patients’ cognitive performance relative to placebo and 2) that the improved cognitive performance would correlate with alterations of the GMV and/or IL-6 level.

## Methods

### Participants and group allocation

For this study, we enrolled 100 consecutive drug-naive patients with first-episode schizophrenia who were treated at the psychiatry departments of Tianjin Fourth Center Hospital, Tianjin Anding Hospital, and Wenzhou Seventh Peoples Hospital, between December 2019 and December 2022. The inclusion criteria were: 1) hospital visitation due to the experience of schizophrenic symptoms for the first time, 2) diagnosis with schizophrenia according to the Structured Clinical Interview for DSM-IV axis 1 disorders (SCID-I) by two senior psychiatrists^77–79^, and 3) no antipsychotic use prior to hospital visitation. The exclusion criteria were: 1) psychotic symptoms not meeting the DSM-IV criteria for schizophrenia, 2) diagnosis of personality disorder, 3) history of a mental disorder induced by physical factors (e.g., severe premenstrual syndrome), 4) history of substance abuse, 5) neurological or other severe physical disease that could influence mental status, 6) MRI contraindication, and 7) refusal to participate in the study. The patients were randomly allocated to two groups receiving placebo and low-dose (250 mg/day) lithium, respectively, adjunct to antipsychotic agents (*n* = 50/group, duration = 24 weeks). All patients received antipsychotic agents to alleviate schizophrenia symptoms. Their liver, thyroid, and kidney functions were monitored weekly. This study was approved by the ethics committees of the three hospitals (Tianjin Fourth Center Hospital IRB no. 2018KY09), and was conducted in compliance with the Declaration of Helsinki. All participants volunteered to take part in this study and provided informed consent.

### Clinical assessment

A research assistant arranged clinical visits and MRI examinations for potentially eligible participants. At the patient visits, two attending psychiatrists collected demographic and clinical information, performed clinical assessments with SCID-I administration, and established schizophrenia diagnoses^80^. The Positive and Negative Syndrome Scale (PANSS)^81^ was used to assess schizophrenic symptom severity and the MCCB^82^ was used to assess cognitive performance.

### IL-6 measurement

Serum samples were collected from the patients and stored at −80 °C. Researchers blinded to participants’ group allocation measured serum IL-6 levels using a Quantikine^®^ immunoassay (R&D Systems, Inc., Minneapolis, MN, USA) according to the manufacturer’s instructions. The intra- and inter-assay coefficients of variability were 1.6–4.2% and 3.3–6.4%, respectively^83–85^.

### MRI examination and image processing

All study participants underwent 3.0-T MRI examination (Discovery MR750; General Electric, Milwaukee, WI, USA) 24 h after clinical assessment. They were given instructions to assure examination effectiveness and safety. T1-weighted (magnetization-prepared rapid acquisition gradient echo) sequences were performed with the following parameters: repetition/echo time (TR/TE), 8.2/3.2 ms; inversion time, 450 ms; flip angle (FA), 12°; field of view (FOV), 256 × 256 mm; matrix, 256 × 256; slice thickness, 1 mm (no gap); and 188 sagittal slices. An experienced clinician screened the images for anatomical abnormalities and artifacts. The T1-weighted images were then processed automatically [with skull dissection, bias field correction, and alignment to Montreal Neurological Institute (MNI) standard space (template 152)] using the Computational Anatomy Toolbox 12 (CAT12, build 1184; Structural Brain Mapping Group, Jena University Hospital, Germany) extension of Statistical Parametric Mapping 12 (Institute of Neurology, University College London, London, UK) in MATLAB (2018b; MathWorks, Inc., Natick, MA, USA). GM/white matter/cerebrospinal fluid segmentation was performed. Group-specific templates were created using a DARTEL algorithm and used as a reference for the non-linear warping and normalization of the segmented images in native space. The CAT12 default parameters and a DARTEL algorithm were used to preprocess the structural MRI data, with bias correction, clarification of tissue type, spatial registration, normalization, and segmentation. For all images, the CAT12 “check data quality using covariance” procedure was executed for quality control.

### GMV calculation

Following segmentation, affine registration to MNI space and nonlinear deformation (using exponentiated Lie algebra) of the GM concentration maps were performed. The data were resampled (cubic voxel size, 3 mm^3^), and voxel-wise GMVs were determined by multiplying the GM map data by the non-linear determinants from spatial normalization. The GM images were smoothed with a 6-mm^3^ full-width-at-half-maximum Gaussian kernel and preprocessed spatially, yielding smoothed maps for statistical analysis. Whole-brain GMV alteration ratios were calculated for the two groups as follows: pretreatment – posttreatment whole-brain GMV/pretreatment whole-brain GMV.

### Statistical analysis

Demographic and clinical variables (age, sex, antipsychotic agent dosage, and educational level) were compared between groups using the SPSS software (version 23.0 for Windows; IBM Corporation, Armonk, NY, USA). Continuous variables are expressed as means ± standard deviations and categorical values are expressed as numbers and percentages. They were compared between groups using repeated-measures analysis of variance, the Mann–Whitney test, Student’s *t* test, and the chi-squared test Bonferroni correction for multiple testing was performed and the significance level was set to *P* < 0.05. The threshold of *P* < 0.05 was also used for cluster-level familywise error–corrected data after the application of an initial cluster-forming threshold of *P* < 0.01. Analysis of covariance was performed to compare the changes in variables from baseline to 24 weeks, adjusted by the baseline levels, between groups. Partial correlation analysis^16,18,26,29,86^ was performed to examine the relationships of cognitive performance to alterations in the whole-brain GMV and IL-6 level and between the latter, with adjustment for age, education level, schizophrenia duration, total PANSS score, and antipsychotic agent dosage (chlorpromazine equivalent).

## Results

### Participant characteristics

In total, 73 patients (37 in the lithium group and 36 in the placebo group) with a mean age of 22.5 ± 2.6 years and mean illness duration of 3.7 ± 1.2 months completed the study. Data from 27 patients were excluded due to the presence of major artifacts or anatomical abnormalities on MR images, failure to meet the CAT12 image quality criteria, or treatment termination. Chi-squared and *t* tests revealed no difference in baseline demographic or clinical characteristics or cognitive performance between the excluded and included patients. The 24 weeks’ cumulative antipsychotic agent dose did not differ significantly between the lithium and placebo groups (105,336.00 ± 8276.25 mg and 106,158.00 ± 8588.287 mg, respectively). The categories of antipsychotic agents and doses are listed in Table 1. No adverse renal event occurred during the study period. The patients’ demographic and baseline clinical characteristics are provided in Table 2.

**Table 1:** Antipsychotic agents used to treat schizophrenia.

Variables	Placebo adjunct antipsychotic agents’ treatment group	Lithium adjunct antipsychotic agents’ treatment group	Antipsychotic agents’ Cumulative dose (mg)
Antipsychotic agents’ name	Number of patients with schizophrenia	Number of patients with schizophrenia	Chlorpromazine equivalent (mg)
Risperidone	15	13	99,878.45 ± 5747.00
Olanzapine	4	2	94,550.11 ± 1259.66
Risperidone adjunct Aripiprazole	3	5	100,377.58 ± 3987.50
Quetiapine	8	10	118,994.63 ± 6959.44
Olanzapine adjunct Aripiprazole	7	6	101,889.47 ± 6259.40

**Table 2:** Baseline patient characteristics (ANCOVA).

	Placebo group	Lithium group	*F*	*P*
Age (years)	22.40 ± 2.12	22.73 ± 1.82	0.452	0.533
Sex (male/female)	12/25	11/25	0.428	0.529
Education (years)	12.54 ± 3.62	14.25 ± 5.20	0.335	0.620
Illness duration (months)	6.22 ± 0.94	4.57 ± 1.45	6.450	<0.001
IL-6 (pg/mL)	7.98 ± 2.00	8.53 ± 1.28	1.890	0.782
PANSS score	80.42 ± 5.62	82.09 ± 8.92	0.223	0.758
MCCB scores				
Processing speed	30.12 ± 2.87	30.60 ± 3.73	1.028	0.066
Attention/vigilance	32.99 ± 6.57	34.69 ± 9.36	0.605	0.471
Working memory	31.22 ± 4.38	32.44 ± 3.70	0.339	0.557
Verbal learning	34.56 ± 3.46	33.39 ± 8.58	0.850	0.100
Visual learning	32.76 ± 9.99	29.35 ± 3.18	1.006	0.091
Reasoning/problem solving	34.77 ± 3.73	30.36 ± 6.87	0.119	0.852
Social cognition	31.54 ± 2.70	33.31 ± 3.69	0.619	0.333
Composite score	35.39 ± 2.84	29.99 ± 1.85	0.822	0.111

### Decreases in PANSS and MCCB scores

PANSS scores decreased significantly after treatment in both groups (Tables 2–5), with no significant difference between groups. MCCB scores in the processing speed and working memory domains improved significantly in both groups; the verbal learning score also improved significantly after treatment in the lithium group, but only processing speed and working memory were significant in placebo group. The alteration rates of MCCB scores in attention/vigilance, working memory, reasoning/problem solving, and social cognition scores changed more in the lithium group than in the placebo group (Table 5). However, no significant within- or between-group difference in the composite MCCB score was observed.

**Table 3:** PANSS and MCCB scores, IL-6 levels, and GMVs in the lithium group (ANCOVA).

Variable	Before treatment	After treatment	*F*	*P*
PANSS	82.09 (8.92)	50.89 (10.23)	33.11	<0.001
IL-6 (pg/mL)	8.53 ± 1.28	3.07 ± 0.98	6.46	0.002
Decrease in whole-brain GMV		0.46%	NA	NA
MCCB scores				
Processing speed	30.60 ± 3.73	33.85 ± 2.33	3.20	0.036
Attention/vigilance	34.69 ± 9.36	35.88 ± 7.95	0.574	0.455
Working memory	32.44 ± 3.70	39.52 ± 5.99	21.05	<0.001
Verbal learning	33.39 ± 8.58	38.00 ± 4.25	4.88	0.029
Visual learning	29.35 ± 3.18	30.99 ± 3.52	0.097	0.985
Reasoning/problem solving	30.36 ± 6.87	33.55 ± 3.56	1.571	0.546
Social cognition	33.31 ± 3.69	34.06 ± 1.88	0.200	0.799
Composite score	29.99 ± 1.85	30.79 ± 5.87	0.555	0.439

Data were analyzed by repeated-measures analysis of variance.

Cumulative 24-week doses of lithium adjunct antipsychotics (chlorpromazine equivalent of antipsychotic agents, 109,200.45 ± 4500.77 mg; lithium 42, 000 mg).

**Table 4:** PANSS and MCCB scores, IL-6 levels, and GMVs in the placebo group (ANCOVA).

Variable	Before treatment	After treatment	*F*	*P*
PANSS score	80.42 (5.62)	48.44 (7.23)	19.532	<0.001
IL-6 (pg/mL)	7.98 ± 2.00	3.5 4 ± 0.69	3.984	0.038
Decrease in whole-brain GMV		1.03%	NA	NA
MCCB scores				
Processing speed	30.12 ± 2.87	32.98 ± 1.47	4.256	0.001
Attention/vigilance	32.99 ± 6.57	32.80 ± 2.95	0.900	0.102
Working memory	31.22 ± 4.38	35.80 ± 2.99	5.693	<0.001
Verbal learning	34.56 ± 3.46	37.00 ± 4.25	6.028	<0.001
Visual learning	32.76 ± 9.99	34.99 ± 3.52	4.377	<0.001
Reasoning/problem solving	34.77 ± 3.73	32.55 ± 3.56	0.589	0.396
Social cognition	31.54 ± 2.70	30.06 ± 3.88	4.822	0.005
Composite score	35.39 ± 2.84	35.00 ± 5.87	0.555	0.439

Data were analyzed by repeated-measures analysis of variance.

Cumulative 24-week antipsychotic dose (chlorpromazine equivalent, 110,376.45 ± 3759.96 mg).

**Table 5:** PANSS and MCCB score, IL-6, and GMV alteration rates in the lithium and placebo groups (ANCOVA).

Variable	Lithium group	Placebo group	*F*	*P*
Decreased PANSS score	31.23 (3.87)	31.00 (5.99)	1.113	0.884
IL-6 reduction ratio	64.01%	55.64%	6.252	<0.001
Decrease in whole-brain GMV	0.456%	1.034%	12.639	<0.001
MCCB scores				
Processing speed	10.62%	9.50%	5.001	0.013
Attention/vigilance	3.43%	−0.58%	1.225	0.074
Working memory	19.90%	6.17%	12.289	<0.001
Verbal learning	4.74%	4.17%	4.233	0.022
Visual learning	5.59%	6.81%	7.255	<0.001
Reasoning/problem solving	10.50%	−6.38%	0.886	0.187
Social cognition	2.25%	−4.69%	7.552	<0.001
Composite score	2.67%	−1.10%	0.938	0.107

### GMV alterations

**Fig. 1:**
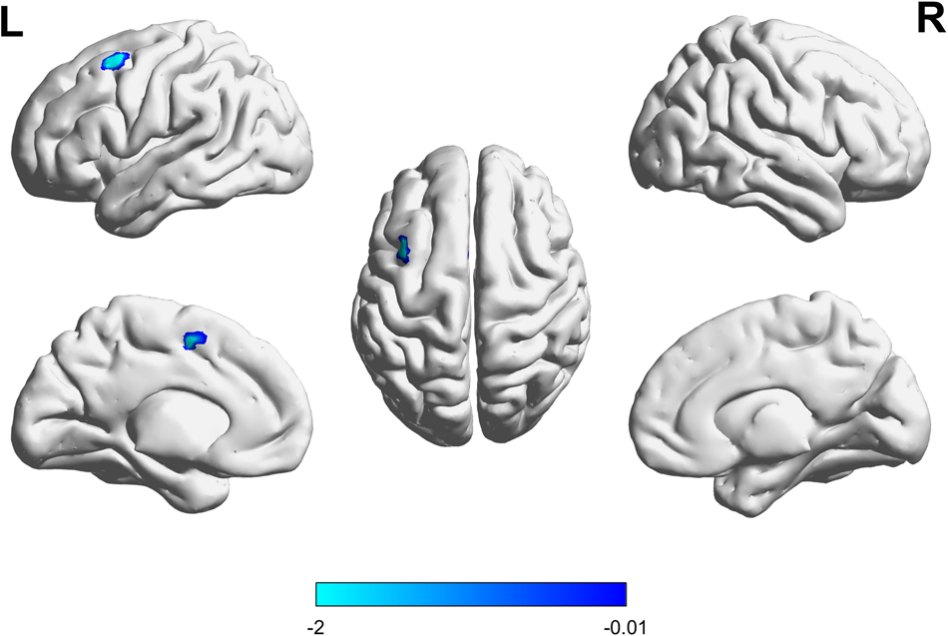
Baseline GMVs (Lithium vs. placebo, *P* = 0.698).

**Fig. 2:**
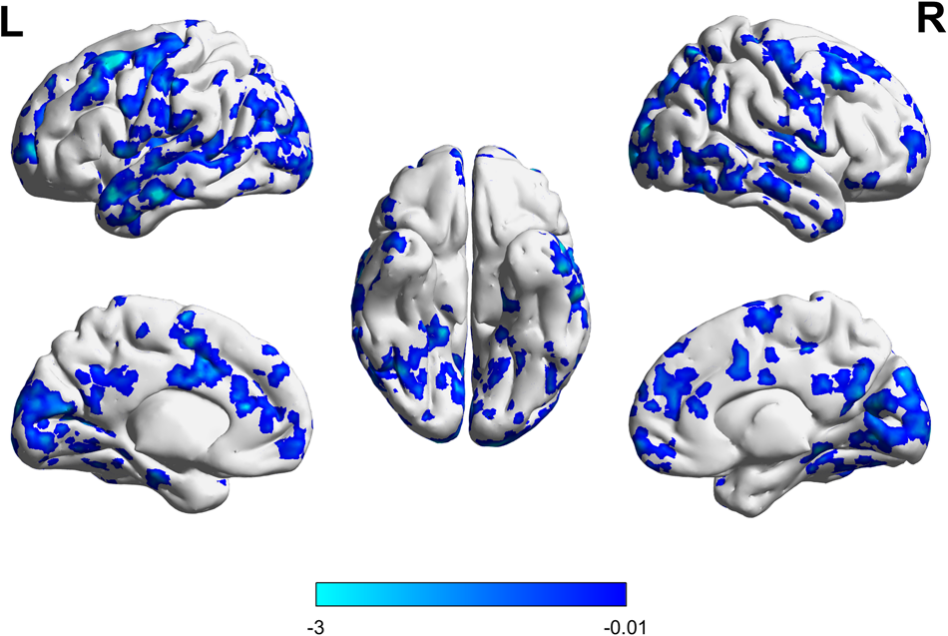
GMVs after 24 weeks of treatment (Lithium vs. placebo, *P* < 0.001).

At baseline, GMVs did not differ significantly between groups (Fig. 1). After 24 weeks of treatment, they were significantly lower in the placebo group than in the lithium group (*P* < 0.001; Fig. 2).

**Fig. 3:**
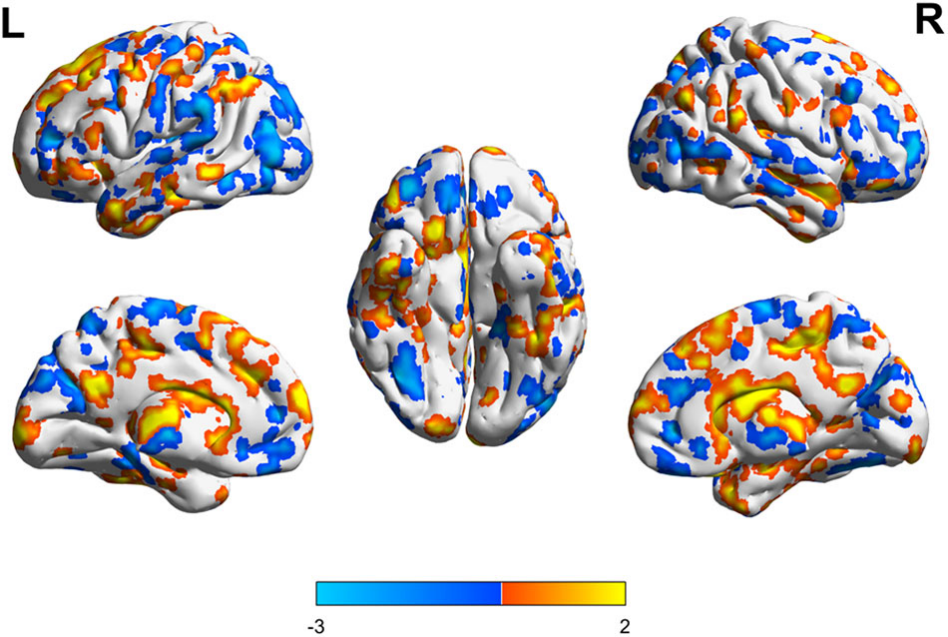
GMV alterations in the lithium group (After vs. before treatment, *P* < 0.001).

**Fig. 4:**
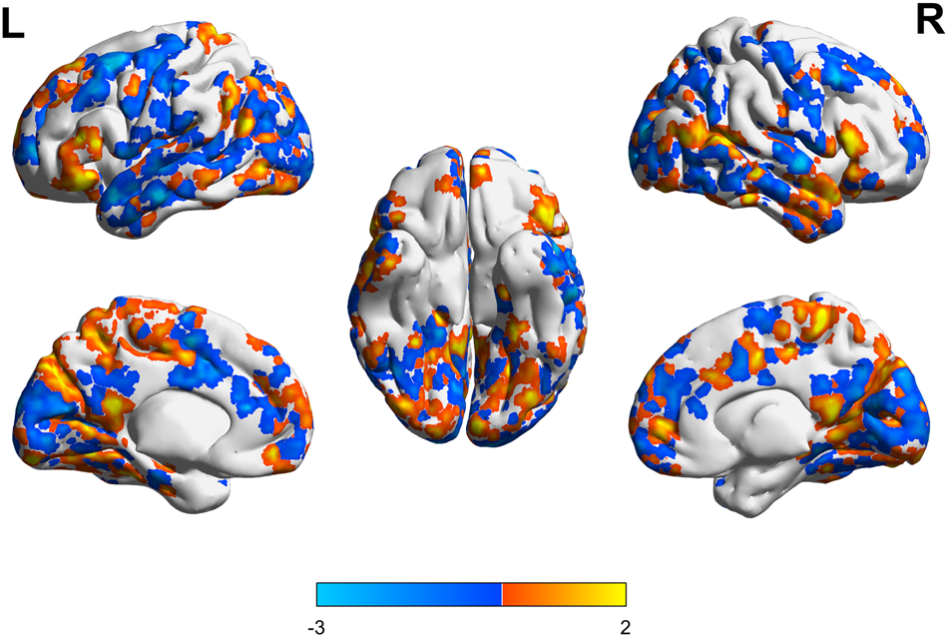
GMV alterations in the placebo group (After vs. before treatment, *P* < 0.001).

Relative to baseline, the lithium group showed GMV reductions mainly in the bilateral occipital, parietal, temporal, and frontal lobes, and GMV increases mainly in the left dorsal frontal, lateral parietal, and occipital lobes and bilateral frontal and parietal lobes and thalamus. This group showed 0.46% whole-brain GMV reduction (*P* < 0.001; Fig. 3). The placebo group showed GMV reductions mainly in the bilateral cingulated gyrus and posterior occipital, temporal, parietal, and frontal lobes and GMV increases mainly in the bilateral prefrontal, left parietal and frontal, and right temporal lobes. This group showed 1.03% whole-brain GMV reduction (*P* < 0.001; Fig. 4).

### IL-6 alterations

The IL-6 level differed significantly between the lithium and placebo groups after treatment (Tables 3 and 4). Compared with baseline, this level decreased significantly by 64.01% in the lithium group and 55.64% in the placebo group; the IL-6 reduction ratio differed significantly, favoring the lithium group (Table 5).

### Correlations among the IL-6 level, whole-brain GMV alterations, and cognitive performance

In the lithium group, the whole-brain GMV reduction ratio correlated with the IL-6 reduction ratio (*r* = −0.19, *P* = 0.040), verbal learning improvement ratio (*r* = −0.17, *P* = 0.025), working memory improvement ratio (*r* = −0.15, *P* = 0.030), and processing speed (*r* = −0.14, *P* = 0.036). The IL-6 reduction ratio correlated with the verbal learning (*r* = −0.30, *P* = 0.031) and working memory (*r* = −0.21, *P* = 0.043) improvement ratios in this group. In the placebo group, the whole-brain GMV reduction ratio correlated only with the working memory improvement ratio (*r* = −0.24, *P* = 0.019) and the IL-6 reduction ratio correlated with the working memory (*r* = −0.17, *P* = 0.022) and verbal learning (*r* = −0.15, *P* = 0.011) improvement ratios.

## Discussion

Our findings showed that a low-dose lithium regimen adjunct to antipsychotic agent use improved some cognitive domains (processing speed, working memory, and verbal learning) in drug-naive patients with first-episode schizophrenia, but resulted in no significant improvement in global cognition. However, our data provide clues for further investigation to identify the ideal treatment strategy to reduce cognitive impairment in patients with schizophrenia.

The PANSS score reductions in both groups indicate that the relief of the patients’ schizophrenic symptoms can be attributed mainly to the antipsychotic agents. Regarding the improvement of the processing speed and working memory in the placebo group, some research has shown that the second-generation antipsychotics used in this study reduce cognitive impairment^87^, such as risperidone, quetiapine, olanzapine and aripiprazole, but results have been inconsistent^88^. Our results suggest that the antipsychotics had protective effects in specific cognition domains, but the lithium group showed more extensive benefits for cognitive performance.

Reductions of the whole-brain GMV and IL-6 level were associated with improved processing speed, working memory, and verbal learning in both groups. The observed whole-brain GMV reduction (despite increases in some brain regions) is consistent with reports that antipsychotic agents can cause brain impairment while alleviating schizophrenic symptoms^89,90^. Given the known positive effects of antipsychotics on cognition, the effect of lithium on cognitive impairment possibly have been synergistic, and lithium did not enhance the alleviation of schizophrenic symptoms. In contrast, low-dose lithium has been found to protect against mild cognitive impairment (MCI) and dementia by increasing the GMV^91,92^. A review showed that lithium increases the GMV by preventing inflammation, oxidative stress, apoptosis, and mitochondrial dysfunction via the phosphatidylinositol-3 (PI3)/Akt/GSK-3β and PI3/Akt/cAMP response element-binding protein/brain-derived neurotrophic factor signaling pathways^93^. Our results are inconsistent with these findings, as regional GMV increases did not overcome the reduction of the whole-brain GMV. The reason for this discrepancy is unclear, and further research on the effects of lithium on the whole-brain GMV is needed.

We observed substantial reductions of the IL-6 level in both groups in this study, consistent with previous findings^26,94,95^. Our finding that this reduction was significantly greater in the lithium group than in the placebo group is consistent with the lithium-induced reduction of the IL-6 level via PI3K/Akt/GSK-3β pathway regulation observed in animal models of cognitive impairment, including mouse models of MCI and dementia^62,96–102^. The mechanism by which lithium reduces cognitive impairment in patients with dementia, MCI, and schizophrenia has not been explored thoroughly, and our results provide a clue for further study.

We cannot fully explain the complex correlations among whole-brain GMV and IL-6 level reductions and cognitive alterations observed in this study. They may be related to mechanisms involving the PI3K/Akt/GSK-3β pathway, homeostasis, the genetic modulation of lithium-induced neural progenitor proliferation, neurotrophic effects, oxidative stress, and/or inflammatory factors^22,26,42–45,62,96–109^. These correlations imply the occurrence of counterintuitive phenomena; for example, adjunct lithium and antipsychotic agent treatments may both induce whole-brain GMV reduction. A decrease in the IL-6 level is usually associated with increased GMV^22,110–112^, but we observed reductions of both. Further research is needed to clarify these complex relationships and underlying mechanisms.

### Strengths and limitations

The main strength of the present study is that it was conducted with drug-naive patients with first-episode schizophrenia, thereby avoiding the potentially biasing influence of medications. This work, however, has several limitations. First, we did not examine the factors underlying the complex correlations among whole-brain GMV and IL-6 level reduction and cognitive alterations. Second, we focused on IL-6 alterations, although other inflammatory factors such as IL-12, IL-1β, tumor necrosis factor-α, and C-reactive protein have crucial effects on the GMV and cognitive performance of patients with schizophrenia, which may explain the inconsistencies between our data and previous findings. Third, the benefits of lithium use in the treatment of schizophrenia have not been fully acknowledged^72,73^; we observed no adverse event in this study, but the benefits and risks of this treatment strategy need to be examined further. Fourth, the second antipsychotic agents used in some cases after the failure of primary agents to alleviate schizophrenic symptoms differed among patients in this study; although the dosages were normalized to chlorpromazine equivalents, different agents target different symptoms of schizophrenia and the diversity of antipsychotic agent regimens may have confounded our study. Finally, whether lithium interacts with antipsychotic agents remains unclear, and potential underlying mechanisms need further study. Further well-designed studies with large samples are needed to clarify these discrepancies.

## Conclusion

The two treatments administered in this study improved specific domains of the cognitive performance of drug-naive patients with first-episode schizophrenia. Low-dose lithium adjunct to antipsychotic agent use had a nearly significant effect on the reduction of cognitive impairment relative to placebo. The patterns of correlation among GMV, and IL-6 level reduction and improved cognitive performances differed between treatment groups.

## Data Availability

The datasets used and analyzed in the current study are available from the corresponding author Chuanjun Zhuo on reasonable request. Anonymized data supporting the findings of this study are available upon reasonable request from the corresponding author for research purposes exclusively.
